# Supplementary material for: Trends of maternal waterpipe, cigarettes, and dual tobacco smoking in Jordan. A decade of lost opportunities
Source: PLoS One. 2021 Jul 9;16(7):e0253655. doi: 10.1371/journal.pone.0253655 (PMC8270187; doi:10.1371/journal.pone.0253655)
Supplement: Attachment — Submitted filename: Reviewer_3_rebuttle.docx [file pone.0253655.s002.docx]

Dear editor,

Thank you for the recent email in regard to reviewer 3 comments. I have not noticed that a third reviewer provided feedback to this article. I apologize for this and kindly ask for accept my response below as instructed.

The updated manuscript has incorporated the initial editorial reviews (copyediting) completed after the first round of review, along with correcting the author’s issue raised by Editor during the second round of review. The third round comments have all been addressed as below.

Best regards,

Khalid Kheirallah, PhD

Reviewers' comments:

Reviewer's Responses to Questions

**Comments to the Author**

1. If the authors have adequately addressed your comments raised in a previous round of review and you feel that this manuscript is now acceptable for publication, you may indicate that here to bypass the “Comments to the Author” section, enter your conflict of interest statement in the “Confidential to Editor” section, and submit your "Accept" recommendation.

Reviewer #3: (No Response)

2. Is the manuscript technically sound, and do the data support the conclusions?

The manuscript must describe a technically sound piece of scientific research with data that supports the conclusions. Experiments must have been conducted rigorously, with appropriate controls, replication, and sample sizes. The conclusions must be drawn appropriately based on the data presented.

Reviewer #3: Yes

3. Has the statistical analysis been performed appropriately and rigorously?

Reviewer #3: Yes

4. Have the authors made all data underlying the findings in their manuscript fully available?

The [PLOS Data policy](http://www.plosone.org/static/policies.action#sharing) requires authors to make all data underlying the findings described in their manuscript fully available without restriction, with rare exception (please refer to the Data Availability Statement in the manuscript PDF file). The data should be provided as part of the manuscript or its supporting information, or deposited to a public repository. For example, in addition to summary statistics, the data points behind means, medians and variance measures should be available. If there are restrictions on publicly sharing data—e.g. participant privacy or use of data from a third party—those must be specified.

Reviewer #3: Yes

5. Is the manuscript presented in an intelligible fashion and written in standard English?

PLOS ONE does not copyedit accepted manuscripts, so the language in submitted articles must be clear, correct, and unambiguous. Any typographical or grammatical errors should be corrected at revision, so please note any specific errors here.

Reviewer #3: Yes

6. Review Comments to the Author

Please use the space provided to explain your answers to the questions above. You may also include additional comments for the author, including concerns about dual publication, research ethics, or publication ethics. (Please upload your review as an attachment if it exceeds 20,000 characters)

**We thank the reviewer for the positive feedback responses above.**

Reviewer #3: I will focus on methods and reporting. Please note that this is the first time I review this paper.
Overall, clear and well written paper, well done.

Major
1) There is no mention of missing data. Were the data 100% complete? if yes then state so. if not, why were multiple imputation models considered?

**Jordan DHS data used interviewer reported responses. There was no missing data that needed imputations. We have noted on other analyses of the Jordan DHS projects that missing data were reported as “missing” and reflected in the total. But this was not an issue with the current variables.**

2) Explain the need for the two modelling approaches. why do you need a time adjusted and an unadjusted model? in other words, what does the unadjusted model add, since the time adjusted model is essential in quantifying any increases over time in the outcomes of interest? and to rephrase again, why don't you drop the time unadjusted model altogether, why is it essential?

**Thank you for this note. We have used the time unadjusted model as a bassline but the focus of the paper was to test changes over the years, for the last decade. It is feasible to actually drop the time-unadjusted model but it also adds strength to the paper to have it as it shows the effect of variables under investigation (overall) for the last decade regardless of the year. The time un-adjusted model was actually similar to the adjusted one; which may indicate that the effect of variables was consistent regardless of year. We hereby ask for the permission to keep it in the manuscript and not to exclude it.**

Minor
1) I struggled to follow the results section in the abstract. it seemed like other outcomes (not previously mentioned) were reported, e.g. "current tobacco use". Please use consistent terminology between the methods and results sections, even if repetitive. also some of the statements are not exactly consistent with the numbers reported e.g. stable when there is an increase and an increase when there is a very large increase.

Thank you for this note. We have edited the manuscript abstract to reflect your comments. We have deleted the term tobacco use as it is clearly stated in the manuscript but not the methods of the abstract. Tobacco use here referred to any type of smoking method (waterpipe or cigarette).

2) discuss the married only status for the respondents as minor or major limitation. In the UK it would be a major limitation since pregnancy is not uncommon outside wedlock. Please explain that this is extremely unlikely in Jordan, as I suspect it is, and possibly reference some evidence to that.

Thank you for this note. Jordan is a conservative society where marriage outside wedlock is punishable by law and puts women at risk of honor killing. This was noted in the study under limitations along with the potential of having limited effects on the results. (1) (2)

3) In the methods section clarify if time was included in the model as a continuous or categorical covariate. Also there is a mention of interaction between socio-economic variables and time. Need clarity whether an interaction term was included (in which case the interpretation of the main effects becomes very difficult if not impossible), or if an interaction was not included (in which case the authors should remove the mention of interaction, since they don't mean statistical interaction, and the term is confusing.

Thank you for this note. We have included year as a categorical variable not continuous as reflected in the model. A note of such was added to the statistical analysis section.

We have also deleted the text referring to the interaction upon your advice as it does not reflect a statistical procedure.

4) Provide a reference for the VIF threshold. I've seen 4 used, and 10 as a more relaxed threshold, but never seen 5 used.

We have reported that none of the VIF values were more than 5. A reference has been identified here and reflected in the paper. It is clearly stated that “Multicollinearity is present when the VIF is higher than 5 to 10 ” (3).

5) Can't have a p-value of 0. when small report as <0.001

Thank you for this note. We have updated the p-values as indicated.

6) restructure tables to include the 95% CI with the estimate. e.g. 1.00 (0.80 to 1.20). I would drop the p-values altogether since they are superfluous.

Tables updated to reflect this note.

7) The paper is lacking in visual evidence, the authors should consider appropriate graphs to better communicate their findings.

We agree with this note and have updated the text to reflect such. New graphs have been added as advised.

7. PLOS authors have the option to publish the peer review history of their article ([what does this mean?](https://journals.plos.org/plosone/s/editorial-and-peer-review-process#loc-peer-review-history)). If published, this will include your full peer review and any attached files.


If you choose “no”, your identity will remain anonymous but your review may still be made public.


**Do you want your identity to be public for this peer review?** For information about this choice, including consent withdrawal, please see our [Privacy Policy](https://www.plos.org/privacy-policy).

Reviewer #3: No

1.	Amnesty International. IMPRISONED WOMEN , STOLEN. POLICING SEX, MARRIAGE AND PREGNANCY IN JORDAN [Internet]. 2019 [cited 2021 May 8]. Available from: www.amnesty.org

2.	Gausman J, Othman A, Dababneh A, Dabobe M, Hamad I, Daas I, et al. A social-ecological examination into the research, policy and health service delivery environment related to early marriage and sexual and gender-based violence among youth in Jordan. BMC Int Health Hum Rights. 2020 Jul 13;20(1).

3.	Kim JH. Multicollinearity and misleading statistical results. Korean J Anesthesiol [Internet]. 2019 Dec 1 [cited 2021 May 8];72(6):558–69. Available from: https://pubmed.ncbi.nlm.nih.gov/31304696/
